# Two Supervised Machine Learning Approaches for Wind Velocity Estimation Using Multi-Rotor Copter Attitude Measurements

**DOI:** 10.3390/s20195638

**Published:** 2020-10-02

**Authors:** David Crowe, Raghava Pamula, Hing Yuet Cheung, Stephan F. J. De Wekker

**Affiliations:** Department of Environmental Sciences, University of Virginia, Charlottesville, VA 22903, USA; rp2zd@virginia.edu (R.P.); xz3ts@virginia.edu (H.Y.C.); sfd3d@virginia.edu (S.F.J.D.W.)

**Keywords:** wind estimation, machine learning, drone attitude, quadcopter, long short-term memory (LSTM), k-nearest neighbors (KNN), unmanned aerial system (UAS), unmanned aerial vehicle (UAV), hovering, horizontal turbulence

## Abstract

In this work we address the adequacy of two machine learning methods to tackle the problem of wind velocity estimation in the lowermost region of the atmosphere using on-board inertial drone data within an outdoor setting. We fed these data, and accompanying wind tower measurements, into a K-nearest neighbor (KNN) algorithm and a long short-term memory (LSTM) neural network to predict future windspeeds, by exploiting the stabilization response of two hovering drones in a wind field. Of the two approaches, we found that LSTM proved to be the most capable supervised learning model during more capricious wind conditions, and made competent windspeed predictions with an average root mean square error of 0.61 m·s^−1^ averaged across two drones, when trained on at least 20 min of flight data. During calmer conditions, a linear regression model demonstrated acceptable performance, but under more variable wind regimes the LSTM performed considerably better than the linear model, and generally comparable to more sophisticated methods. Our approach departs from other multi-rotor-based windspeed estimation schemes by circumventing the use of complex and specific dynamic models, to instead directly learn the relationship between drone attitude and fluctuating windspeeds. This exhibits utility in a range of otherwise prohibitive environments, like mountainous terrain or off-shore sites.

## 1. Introduction

The versatility and ubiquity of unmanned aerial vehicles (hereafter referred to as drone) afford an accessible method to derive wind conditions above the surface. While existing work has endeavored to accomplish this problem, either the experimentations were conducted in a controlled indoor environment, or otherwise relied on complex mathematical representations of vehicular dynamics, which are less extensible to other unmanned aircraft. Our work appraises the windspeed prediction accuracy of two machine learning (ML) algorithms, trained with high temporal frequency accelerometer-derived attitude data from two separate drones, across two measurement days and validated against recorded wind measurements from a nearby 3D sonic anemometer. In addition to windspeed estimation, we provide a first approximation of horizontal turbulence characterization using windspeed variances, as in [1,2].

While more direct methods of drone-based wind measurements exist [3,4], they oftentimes rely on expensive sensors, which are not only subject to disturbances from the drone propellers and orientation, but also limit flight duration by more rapidly depleting the battery from the additional weight. Other approaches which confronted the problem at hand required thorough parameterizations of drone dynamics to develop rigid body models and non-linear relationships between drone attitude and windspeed [5,6,7,8]. Such methods, while shown to be effective, are less extendable to other drone models than our proposed methodology, which eliminates the need for complex mathematical representations by instead learning the wind-attitude relationship directly from available attitude data. Work in [9] established the feasibility of using k-nearest neighbors (KNN) for rotary-wing-based windspeed estimation. However, speeds in their indoor experimentation did not exceed 3.07 m·s^−1^, which constrains the applicability and representativeness of their findings. Similarly, the authors in [10] developed a drone-based long short-term memory (LSTM) framework to predict windspeeds, but in a virtual environment, using wind data produced from both a Dryden wind turbulence model over flat terrain, and large eddy simulations. Our work builds upon these theoretical foundations, and extends the findings to consider a real-world topographically-rich environment, which more robustly supports the utility of the supervised learning methods in applied settings.

Among the most difficult phenomenon to predict is atmospheric turbulence. For decades micrometeorologists have faced a closure problem which precludes accurate weather prediction beyond a few days’ time. Such models suffer from uncertainties, in part because of the parameterization of unknown elements like turbulence [11]. By improving our understanding of turbulent motion, where and how it arises, numerical weather prediction models may improve upon their turbulence parameterization schemes which in turn provide more accurate weather forecasting. In addition to this motivation, the transport of chemical pollutants is in part governed by wind and turbulence. The oftentimes tempestuous motion arises from a variety of potentially compounding factors including updrafts formed from localized surface heating or collisions between cold and warm air masses. Heterogeneous terrain poses additional complications in characterizing wind and turbulence regimes through mechanisms like orographic lifting and drainage flow. By leveraging the versatility of drones, such numerical models may benefit from the inclusion of data procured from otherwise unavailable locations. Moreover, windspeed and direction measurements are a key element in determining the viability of offshore wind farm locations [12,13]. Conventional meteorological masts are costly and challenging to setup in these regions, given the necessary spatial breadth of measurements. Our approach offers an alternative cost-effective in-situ windspeed collection system to provide such data in offshore environments. To demonstrate commercial viability, such environments should have an average annual wind speed of 7 m·s^−1^ or greater at 90 m [14], which is within the flight capacity of the hobby-grade drones presented in this work. In addition to the speed and directional data, approximating the turbulence intensity can help initialize simulations that estimate wind turbine fatigue load.

The ensuing sections of this letter are structured as such: Section 2 presents the environment and equipment used in experimentation, and provides the conceptual framework behind the two proposed supervised ML methods, in addition to a brief overview of procedures to characterize turbulence. Section 3 demonstrates the resulting effectiveness of both approaches, as compared with a simple linear regression model across two days of flying, to identify windspeed regimes where our methods are shown to be more suitable. Finally, Section 4 interprets the results, remarks on the generalizability of the findings, acknowledges limitations and considerations, and suggests avenues to advance the state of this research.

## 2. Materials and Methods

### 2.1. Measurement Site and Equipment

Data used to train and validate the models were collected near the base of the Blue Ridge Mountains at Innisfree Village, located within central Virginia on 26 March 2020 and 3 June 2020. The valley contains complex terrain, and lies 312 m above mean sea level (ASL), with adjacent ridges exceeding 700 m in height [15]. The conditions during both days were partly cloudy, and flights were conducted shortly after noon. Suitable flying days were selected to capture a wide range of wind velocities up to around 7 m·s^−1^. To collect drone attitude data, a 1.5 kg 3DR Solo (3D Robotics Inc., Berkeley, CA, USA), and a 0.74 kg DJI Mavic Pro (DJI Sciences and Technologies Ltd., Shenzhen, China) were used to collect 3D accelerometer and accompanying roll and pitch data. A WindMaster 3D sonic anemometer from (Gill Instruments Ltd., Lymington, UK) was mounted 10 m atop a weather tower and recorded 10 Hz windspeed and direction data at a resolution of 0.01 m s^−1^ with a reported accuracy of <1.5% root-mean-square error at 12 m·s^−1^ [16]. As the vertical wind component was approximately 2 orders of magnitude lower than the horizontal components, we used the horizontal components to compute windspeed and find their variances as a proxy for turbulence.

### 2.2. Data Collection and Processing Procedures

**Figure 1:**
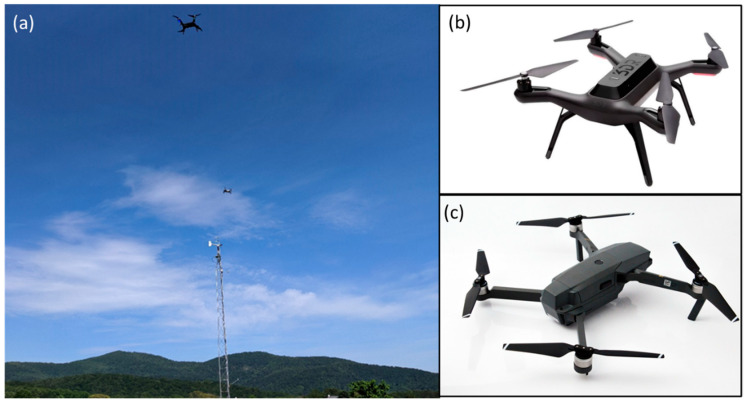
Field setup (**a**) of the 3DR Solo (**b**) and DJI Mavic Pro (**c**) drones in relation to the sonic anemometer. The Mavic was set to hover approximately 6 to 8 m from the anemometer and the Solo an additional 7 m away. Both drone images are used under the Creative Commons Attribution-Share Alike 4.0 International license.

For each day, three flights were conducted producing approximately 37 min of hovering drone data and corresponding windspeed data per day. For every flight, the Mavic was stationed 6 to 8 m west of the anemometer and the Solo flew approximately 7 m from the Mavic, farther west. Both drones were flown simultaneously, and generally hovered in place facing as close to magnetic north as possible at about the same elevation of the 10 m tower top for 10 to 13 min each flight. Drone data from the Mavic were natively stored onboard, while the Solo was equipped with a Pixhawk 2.1 Green Cube (ProfiCNC, Moolap, Australia) which facilitated accurate attitude measurements. All data were resampled from 10–30 Hz to 1 Hz for model training and prediction, by averaging all available sub-second data into the corresponding second bin. Using higher frequency data presented complications, due likely in part to small temporal discrepancies between the attitude measurements and recorded windspeeds, which introduced substantial error throughout the training process. The standard flight configuration and drone craft are shown in Figure 1.

Since the GPS time-keeping on the drones differed from the time on the anemometer, and because both drones were flown at a distance from the anemometer to mitigate wake effects or data contamination, an offset for each flight was found to remedy the phase incongruities of each time series. To aid in identifying the optimal offset, the dynamic time warping (DTW) Python library [17] supplied both the windspeed and the drone tilt. The tilt, α, combines roll, μ, and pitch, δ, data into a single variable using the relationship given in [3]:

(1) $$\alpha =\;\cos^{-1}\left(cos\;\mu \;cos\;\delta \right)\;\;$$

This method provided an objective measure of the similarity between both time series to optimally align the data, as this is a crucial step in training the supervised models to make accurate time series-dependent predictions. Beyond the roll and pitch, the 3-dimensional accelerometer data from both drones were leveraged to train and make predictions. To optimize model performance, the drone data input underwent a commonly used standardization scheme to scale all features in the same fashion as given by:

(2) $$x\prime \;=\;\frac{x\;-\;\bar{x}}{\sigma }$$

where *x* is the original feature vector (roll, pitch, and XYZ accelerometer data), $\bar{x}$ is the mean of the vector, and σ is the standard deviation.

### 2.3. Machine Learning Models

Supervised machine learning (ML) algorithms require ground truth data (or labels) to train capable prediction-making models, which contrasts with unsupervised learning methods that instead seek to discover emergent patterns within input data. This research proposes two supervised ML models using drone inertial and sonic anemometer data which have been made publicly available and may be found in the Supplementary Materials. This section will give a cursory overview of the two supervised models used in this work.

#### 2.3.1. K-Nearest Neighbors (KNN)

**Figure 2:**
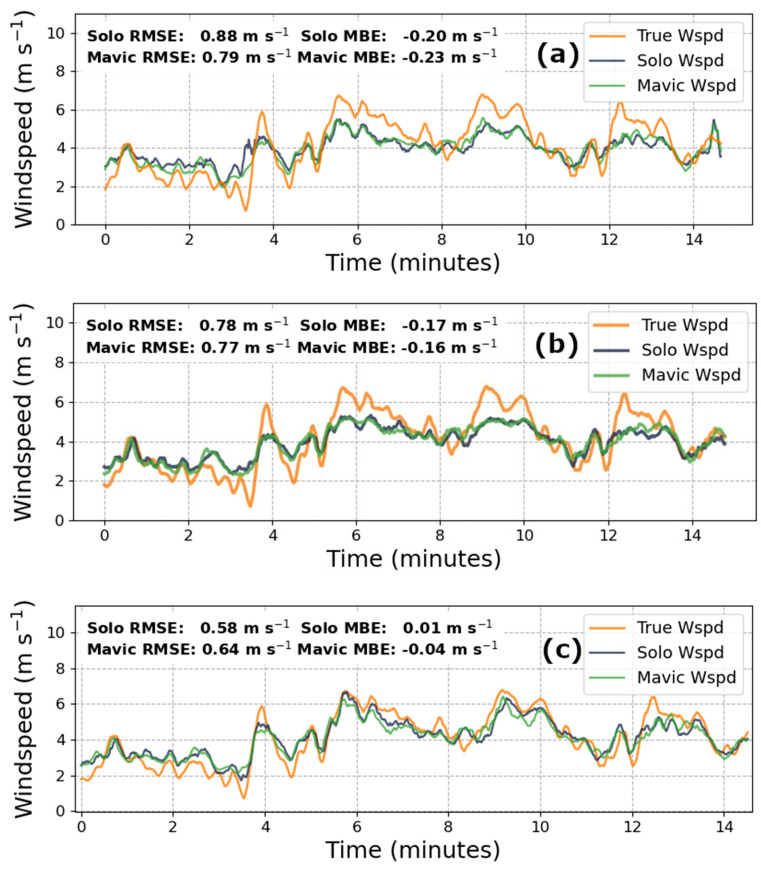
Cross-model comparison of prediction accuracy for the three concatenated March flights when trained on 22 min of data, where (**a**) is the linear regression model (**b**) is the K-nearest neighbor (KNN) algorithm with K = 11 for Solo data and K = 18 for Mavic data, and (**c**) is the long short-term memory (LSTM) neural network.

**Figure 3:**
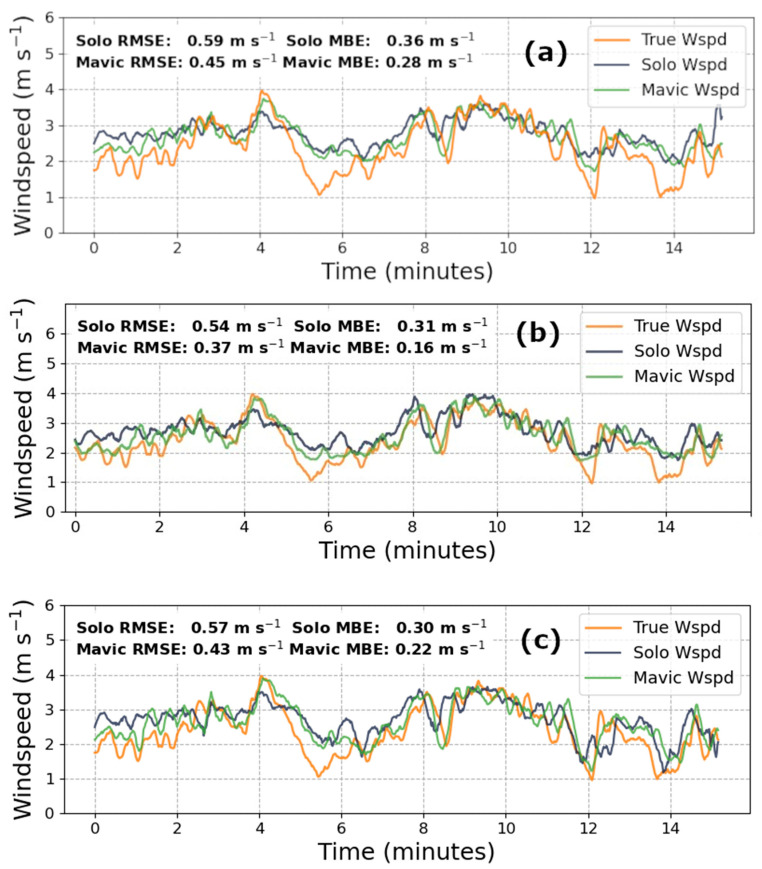
Cross-model comparison of prediction accuracy for the three concatenated June flights when trained on 23 min of data, where (**a**) is the linear regression model (**b**) is the KNN algorithm with K = 40 for Solo data and K = 70 for Mavic data, and (**c**) is the LSTM neural network prediction.

The basic premise of the KNN algorithm clusters input data based on a distance metric (in our case Euclidian), and classifies new data based on its proximity to existing groupings. The “K” parameter determines how many adjacent data (i.e., neighbors) are considered before assigning this cluster’s label to the data in question. For our purposes, K was chosen for each dataset from a range of 2 to 100 by identifying the K that produced the lowest root mean square error for the validation set, as reported in Figure 2b and Figure 3b. The primary advantage of employing KNNs is the rapid speed of model training and prediction-making, which supports its use as a resource-efficient and near real-time windspeed measurement technique.

#### 2.3.2. Long Short-Term Memory (LSTM)

LSTMs are a variant of the greatly successful recurrent neural network (RNN), which excels at making predictions from sequential data. The basic operation of an RNN iteratively computes and compares a prediction from a state measurement against ground truth data (in our case drone-based windspeed predictions and anemometer windspeed measurements) using a loss or cost function to find an error value. This value is then used in backpropagation to continuously adjust internal weights and biases in the model before arriving at a minimum error. The input of a given RNN unit is not only dependent upon new information but also on the output from the previous timesteps, hence the recurrent nature. This contextual information is contained within a hidden state of the recurrent network, and is responsible for maintaining long-term dependencies. The hidden state $h_{t}$ of an RNN can be modeled as:

(3) $$h_{t}=\lambda \left(Wx_{t}+\;Uh_{t-1}\right)$$

where $x_{t}$ is the current input vector, W is a weight matrix, and U is the hidden-state or transition matrix. The matrices work in conjunction to determine the significance of the new input and the prior hidden state. The sum of both the weighted input and hidden state is transformed by λ, an activation function that condenses the values into a more usable space (usually between −1 and 1 or 0 and 1). A more in-depth excursion into the machinations of RNNs may be found in [18,19]. RNNs using multiple layers may suffer from the vanishing gradient problem, where the model fails to appropriately learn parameters of the primary layers, which results in the model’s inability to make effective use of data from earlier in the sequence. To combat this issue, the LSTM contains additional gates that employ non-linear activation functions that allow the model to selectively decide which information to retain and which to forget. Several successful use cases for RNNs and LSTMs include: natural language processing; machine translation; music composition; speech recognition; and image captioning [19].

The LSTM model used in this work was built using TensorFlow’s Keras library [20]. Our model consisted of four hidden layers with 420, 180, 90, and 48 nodes in each layer, and a rectified linear unit (ReLU) activation function, to curtail vanishing gradients during backpropagation. The final LSTM model was trained across 100 epochs, with batch sizes of 16, and optimized using an RMSprop algorithm with a learning rate of 3 × 10^−5^. The only distinction between the models used in the March and June flights is that the former included a dropout value of 0.96 within the third layer (with 90 nodes) to help combat overfitting. This architecture was chosen by exhaustively evaluating different combinations of layer counts, node densities, dropout rates, and optimizers, to identify a design that performed satisfactorily across all trials. Once the model has been trained on the allocated data, windspeed predictions are made for each individual state measurement of inertial information. The shape of the input to the first layer is therefore the accelerometer data and accompanying roll and pitch values, while the output is a single windspeed prediction based upon these data.

### 2.4. Model Metrics

To appraise the efficacy of our approach, the mean bias error (MBE) and root mean squared error (RMSE) were found for both the KNN and the LSTM models, and compared with those found from a linear regression model. The RMSE is a commonly used metric found in related literature, and measures the average magnitude of the errors, while the MBE helps determine whether the model overestimates or underestimates its predictions, and is defined as:

(4) $$MBE=\frac{1}{n}\overset{n}{\underset{i=1}{\sum }}P\left(i\right)\;-\;O\left(i\right)$$

where P  is the predicted windspeed and O is the ground truth. By this definition, positive values of MBE indicate overestimated predictions, while negative MBEs imply the model underestimates the windspeed. RMSE is defined as the square root of the average squared differences between the predicted and observed values:

(5) $$RMSE=\sqrt{\frac{1}{n}\overset{n}{\underset{i=1}{\sum }}{\left(P\left(i\right)\;-\;O\left(i\right)\right)}^{2}}$$

### 2.5. Turbulence Characterization

For a variety of applications, e.g., air pollutant dispersal, turbulence model evaluation, and assessment of a region’s wind farm potential, it is important to know the turbulence variance for each horizontal component, u and v, which respectively represent the zonal (towards the east) and meridional (towards the north) speeds [2]. To do this, the meteorological wind direction φ is first found by adding 180° to the wind vector azimuth θ (the direction in which the wind is moving), as in Equation (7). The wind vector azimuth was computed using the drone’s roll and pitch via Equation (6), as in [3]. As we maintained that the drones were flying facing north, the yaw remained essentially zero throughout data acquisition. With φ in hand, the windspeed ϑ can be decomposed into the constituent u and v vectors, following Equations (8) and (9).

(6) $$\theta \;=\;arctan\left(\frac{-\sin\mu \cos\delta }{\cos\mu \sin\delta }\right)\;+\;180{}^{\circ}$$

(7) φ =θ+ 180°

(8) $$u\;=\;-\vartheta \;\times \;\sin\left(\frac{\pi }{180}\times \varphi \right)$$

(9) $$v=-\vartheta \;\times \;\cos\left(\frac{\pi }{180}\times \varphi \right)$$

The variances for both components were computed using 1 Hz data, and compared against the variances of corresponding recorded wind data for both days.

## 3. Results

### 3.1. WindSpeed Estimation

Here we compare the results of two separate ML models in conjunction with a linear regression model to better assess the performance gains. For all models, a 10 s moving average filter was applied to each time series before computing the established metrics. To perform fair comparisons, the same training and validation ratio of 60:40 was used across all models for both days so that each model was informed with precisely the same amount of information. Although both machine learning models improved their performance when more training was available, we found this ratio provided the greatest amount of validation data, and hence fewer training data, while sustaining a reasonable amount of error.

The windspeed prediction performances for each model, and both drones, across the three concatenated March flights are displayed in Figure 2. In total, approximately 37 min of data were available for use from this flight day, of which 22 were reserved for training, and the remaining 15 for validation. The range of recorded windspeeds during the training period spanned 1.78 m·s^−1^ to 6.46 m·s^−1^ while the validation data exhibited a domain of 0.71 m·s^−1^ to 6.78 m·s^−1^. The mean windspeeds for the respective periods were 4.09 m·s^−1^ and 3.89 m·s^−1^.

Figure 3 displays the performance of the three models on the concatenated June flight data. Atmospheric conditions in June provided less tempestuous wind for testing. For this day, roughly 38 min of data were available for use, with 23 available for training, and 15 for validation. The windspeeds in the training data ranged from 1.15 m s^−1^ to 6.02 m s^−1^ owing to a brief gust that occurred a few minutes into hovering, with an average windspeed of 2.40 m s^−1^. Windspeeds in the validation set spanned 0.96 m s^−1^ to 3.97 m s^−1^, and contained a mean speed of 3.05 m s^−1^.

Table 1 and Table 2 summarize the RMSE and MBE found from the predicted windspeed, from 15 min of validation data across each model and drone pair from both flight days.

In June, during more quiescent conditions, the RMSE and MBE of the Mavic KNN model showed a 37%, and 43%, improvement, respectively. Interestingly, the June Solo RMSE was 27% greater than the linear model, while the MBE improved by 17%. During the March flights, when the winds were more variable, both the Mavic and Solo KNN model offered an approximately 3% improvement in RMSE, and a 30% reduction in bias. Most importantly, the LSTM models for the Mavic and Solo, respectively, demonstrated a 19% and 34% improvement in RMSE (which are values similar to those seen in related literature), and an 82% and 95% decrease in the MBE. While the MBE of the KNN models during the March and June flights demonstrated a consistent underestimation and overestimation of windspeeds, respectively, these metrics do not exceed an absolute value of −0.17 m·s^−1^ in March and 0.32 m·s^−1^ in June. The Solo’s MBE for June flights remained between 0.30 m·s^−1^ and 0.32 m·s^−1^, which contrasts with the improvement reported by the Mavic predictions. A possible explanation for this disparity may lie in the procedural differences across each drone’s flight controller which, under calmer conditions, may have introduced comparatively larger errors in the inertial measurements while stabilizing the quadcopter. For March data during more variable winds, the LSTM model predictions maintained an absolute MBE of less than 0.04 m·s^−1^ across both drones. Even in the worst cases, the MBEs from the ML models are still well within the range of results reported in related literature.

### 3.2. Turbulence Characterization

**Figure 4:**
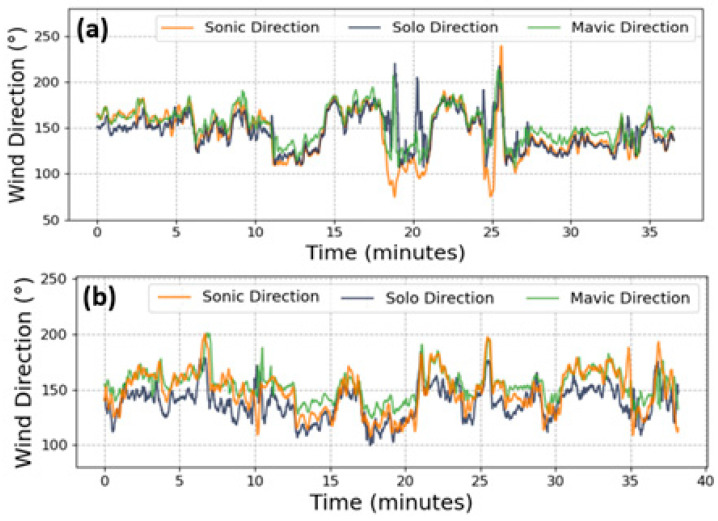
March (**a**) and June (**b**) wind direction computed using roll and pitch data according to Equation (6) for the entirety of both datasets. Data underwent a 10 s moving average for the purposes of demonstration alone; to compute the turbulence quantities 1 Hz data were used.

To decompose the predicted windspeed into the necessary horizontal components, the wind direction was first computed according to Equation (6) for both drones. A comparison of the drone-based wind directions with ground truth wind direction measurements from the sonic anemometer for the three concatenated flights per day is presented in Figure 4. The 1 Hz data were used to calculate the turbulence measures.

**Figure 5:**
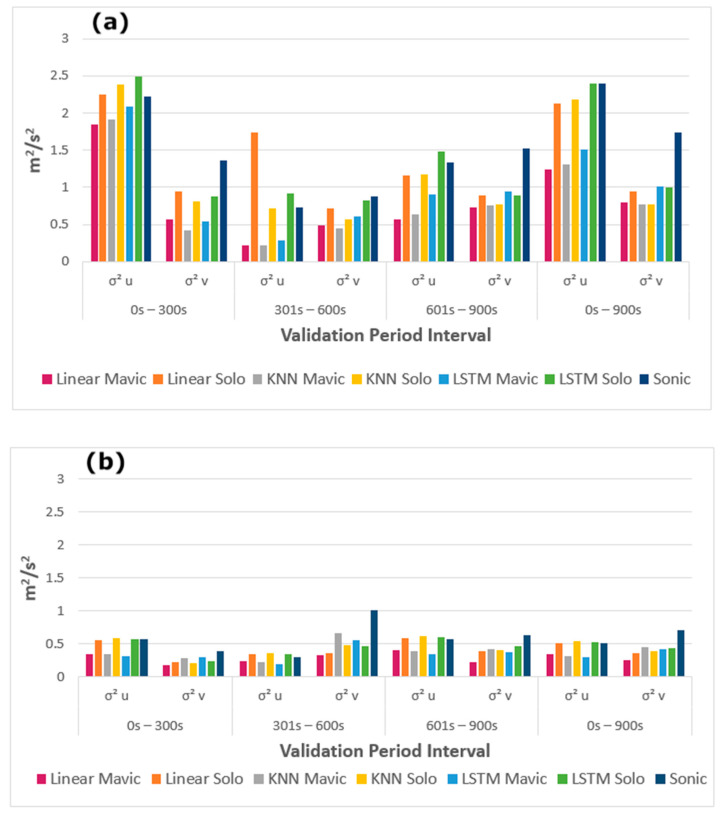
March (**a**) and June (**b**) horizontal windspeed variances from each drone-model pair data, and from the sonic anemometer, across three five-minute periods and throughout the 15-min validation data.

To address the suitability of using predicted windspeeds and inertial drone data as a means to characterize turbulence, we compared predicted and ground truth horizontal component-wise windspeed variances for both days according to Equations (6)–(9), across three five-minute time intervals in addition to the entire 15 min period as shown in Figure 5.

The turbulence variances from the predicted windspeeds were generally underestimated, owing in large part to the penalties associated with highly variable wind speeds during the model training process. From Figure 5, the differences in windspeed variability across both flight days become apparent. Since the winds in March were more variable than those in June, we presume the models’ wider exposure to wind speeds bolstered its predictions by expanding the gamut of training data to more robustly estimate using new data.

## 4. Discussion

We have demonstrated the potential of two machine learning approaches for inferring the wind velocity under a range of realistic environmental wind regimes. Under calmer conditions (i.e., during the June flights) the linear model appears to perform comparably to the ML approaches. However, when the variance of the windspeed rises, the relationship between the drone’s stabilization and windspeeds grows increasingly non-linear, as seen in its March predictions. In such cases, the KNN algorithm offers a marginal improvement, while the LSTM exhibits a greater utility for this wind case. One of the core objectives of this work sought to minimize the amount of training data necessary to produce competent predictions. We found that despite adding more data to the training set, the KNN and LSTM models did not experience a substantial reduction in their RMSE and MBE. Thus, a 60:40 split was used throughout the trials to partition training and validation data. Still, using smaller amounts of training data (of at least 12 min) was shown to produce capable predictions. We also found that the LSTM’s effectiveness was highly sensitive to the number of nodes in the final layer, and the dropout value used in the third layer. It should be noted that for the June Mavic flights, only roll and pitch data were available and used in training. For all other flights, the three-dimensional accelerometer data were also considered. Their exclusion did not substantially alter model performance.

One of the most significant factors that precluded ideal predictions was the differing temporal synchronization between the drone attitude data and windspeeds. Despite being spatially consistent, the distance between the drone and tower may not have always been sufficient to determine the time disparity between the two data. In the event of a wind gust, and depending on its direction, for example, an eddy-front may have more rapidly reached the drone before passing the anemometer (or vice versa), which variably disarranged the attitude relationship with the wind velocity. While higher resolution data are preferred to better capture the ephemeral eddies, we resampled all data to 1 Hz to simplify the alignment procedure between drone data and recorded windspeeds, and mitigate complications during the model training process, which arose when using higher resolution data. These complications were likely exacerbated by the variable synchronicity between drone inertial measurements and ground truth measurements. To combat this, a dynamic offset, instead of the per-flight static offset used herein, for each data point, incorporating the recorded windspeed and direction data, could better ensure that both time series are harmonized. Alternatively, flights could be performed on a day with persistent flows from a single direction, or drones may be flown on opposing sides of the anemometer. One explanation behind the differences in drone-based and observed horizontal windspeed variances is that the drones may have at times not been perfectly pointed north. Still, our appraisal of the drones’ ability to compute the wind direction in addition to the windspeed predictions supports its potential for analyzing longitudinal and lateral turbulence variances. However, it must be stated that the models tended to make less variable predictions than the data encountered during the training phase, which resulted in lower windspeed variances across both flight days, as seen in Figure 5. This persistent underestimation is, in part, a symptom of the drone’s stabilization response to the rapid wind fluctuations, which actively attempts to safely maintain a steady attitude; the directional disparities between the drones and ground truth; and of the imperfect windspeed predictions. Most importantly, however, the comparatively lower variances can be explained in large part by the inherent reduction in highly variable windspeed predictions, as these are likely to be more severely penalized during the training process. To potentially combat this, higher frequency data may be used to introduce greater natural variability, although it should be noted that the alignment of the drone data and recorded windspeeds will become more sensitive and may result in an overall reduction of predictive accuracy. Despite this, the drones can competently compute wind directions using 1 Hz roll and pitch data (Figure 4), which alone is a valuable feature of using such vehicles in meteorology.

Given the transient nature of the atmosphere, and the 15-min length of the validation periods, we partitioned the variance calculations into five-minute bins. To more properly characterize turbulence, all three directional components should be considered during analysis. Our focus on horizontal wind speeds did not allow us to estimate the vertical velocity which, during our field tests, were two orders of magnitude smaller than the horizontal velocity components. Therefore, we were calculated the horizontal wind variances as a first approximation of turbulence. To possibly estimate vertical wind perturbations, and hence develop a more coherent picture of turbulence, days with greater convection or buoyancy should be chosen to gather data, in order to provide the model with more salient vertical wind information during training.

While hobby-grade drones like the ones presented in this work can capably fly in windspeeds of up to approximately 10–13 m·s^−1^, the largest risk in applying this method under high intensity wind regimes, like those often found in offshore wind turbine sites, would be during take-off and landing situations. Higher tier quadcopters may fare better under such conditions, which should offer more truthful predictions.

One potentially fruitful extension of our approach would be to probe the wind structure of the lowermost boundary layer via drone ascent, as in [5], to reveal the columnal properties of transient atmospheric flows. Such vertical profiles would offer valuable insights, such as wind shear aloft, to unveil a deeper understanding of the dynamic nature of an ever-changing atmosphere. Another possible application could implement a multi-agent swarm of identical drones in an optimized configuration, to probe the lower atmosphere instantaneously at a higher spatial resolution. Yet another avenue worth exploring would be to assess the effectiveness of off-the-shelf Bluetooth accelerometers mounted onboard hobbyist-grade drones, whose flight controllers may not necessarily make attitude and accelerometer data available to the user. Such use of easily accessible accelerometers could potentially open up the potential to predict and report windspeeds in near real-time through crowdsourcing initiatives.

## 5. Conclusions

Ultimately, while our methods build upon existing theoretical frameworks proposed by [9,10], to the best of our knowledge this work presents the first scientific investigation of a real-world application using machine learning methods to accurately predict windspeeds using onboard drone attitude and inertial data. During placid periods of wind activity, we found that the linear model performed on par with the KNN algorithm and LSTM neural network. However, under more variable wind conditions we have shown that the LSTM outperforms the linear model and KNN algorithm, and offers comparable performance to more sophisticated and vehicle-specific techniques [3,4,5,6,7], which are nontrivial to implement, and less extensible to other unmanned aircraft. Our approach circumvents the need to develop such specialized tools by instead exploiting the equilibrating response of a hovering drone in a wind field to directly learn the relationship between drone attitude response and windspeed. This methodology offers a cost-effective alternative for sampling windspeeds in a variety of logistically challenging domains, like potential offshore wind farm sites, or within topographically heterogenous terrain.

## Figures and Tables

**Table 1:** Comparison of root mean square error (RMSE) and mean bias error (MBE) across each drone and model type for March flights.

Model and Drone	RMSE (m·s^−1^)	MBE (m·s^−1^)
Linear Mavic	0.79	−0.23
Linear Solo	0.88	−0.20
KNN Mavic	0.77	−0.16
KNN Solo	0.78	−0.17
LSTM Mavic	0.64	−0.04
LSTM Solo	0.58	0.01

**Table 2:** As in Table 1 now for June flights.

Model and Drone	RMSE (m·s^−1^)	MBE (m·s^−1^)
Linear Mavic	0.59	0.28
Linear Solo	0.45	0.36
KNN Mavic	0.37	0.16
KNN Solo	0.54	0.32
LSTM Mavic	0.43	0.22
LSTM Solo	0.57	0.30

## Supplementary Materials

The drone inertial and anemometer data used for our analysis may be found at: https://github.com/asthecroweflies/drone_ml_wind_estimation.
