# Supplementary figures and images for: Two Supervised Machine Learning Approaches for Wind Velocity Estimation Using Multi-Rotor Copter Attitude Measurements
Source: Sensors (Basel). 2020 Oct 2;20(19):5638. doi: 10.3390/s20195638 (PMC7583910; doi:10.3390/s20195638)

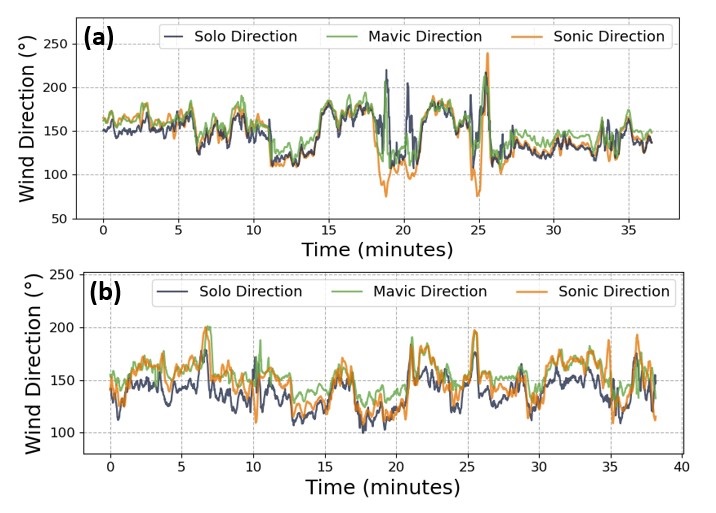

Supplement: Supplementary file 1 [file sensors-20-05638-s001.zip › flight_directions.jpg]

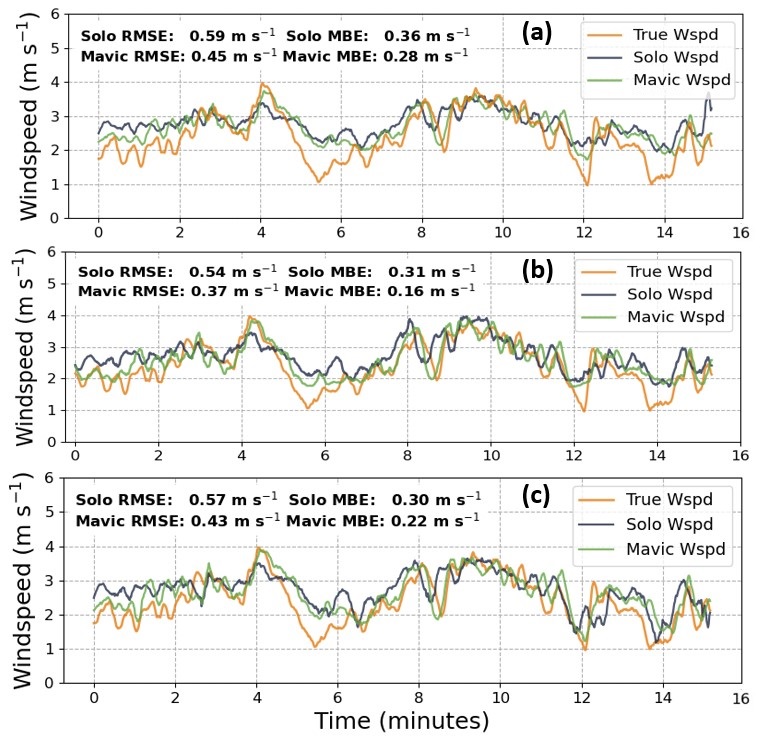

Supplement: Supplementary file 1 [file sensors-20-05638-s001.zip › june_flights.jpg]

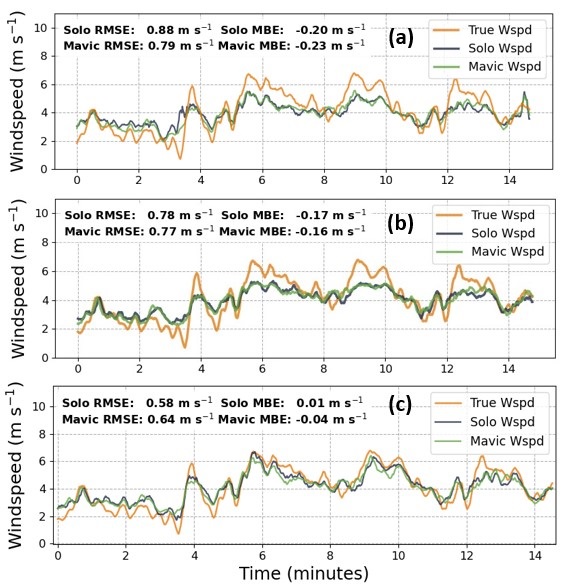

Supplement: Supplementary file 1 [file sensors-20-05638-s001.zip › march_flights.jpg]
